# Plasma metabolomics profiles in rats with acute traumatic brain injury

**DOI:** 10.1371/journal.pone.0182025

**Published:** 2017-08-03

**Authors:** Fei Zheng, Zi-An Xia, Yi-Fu Zeng, Jie-Kun Luo, Peng Sun, Han-Jin Cui, Yang Wang, Tao Tang, Yan-Tao Zhou

**Affiliations:** 1 College of Electrical and Information Engineering, Hunan University, Changsha, China; 2 Department of Integrated Traditional Chinese and Western Medicine, Laboratory of Ethnopharmacology, Xiangya Hospital, Central South University, Changsha, China; 3 College of Pharmacy, Shandong University of Traditional Chinese Medicine, Jinan, China; University of Florida, UNITED STATES

## Abstract

Traumatic brain injury (TBI) is a major cause of mortality and disability worldwide. We validated the utility of plasma metabolomics analysis in the clinical diagnosis of acute TBI in a rat model of controlled cortical impact (CCI) using gas chromatography/mass spectrometry (GC/MS). Thirty Sprague-Dawley rats were randomly divided into two groups of 15 rats each: the CCI group and sham group. Blood samples were obtained from the rats within the first 24 h after TBI injury. GC/MS measurements were performed to evaluate the profile of acute TBI-induced metabolic changes, resulting in the identification of 45 metabolites in plasma. Principal component analysis, partial least squares-discriminant analysis, orthogonal partial least square discriminant analysis using hierarchical clustering and univariate/multivariate analyses revealed clear differences in the plasma metabolome between the acute CCI group and the sham group. CCI induced distinctive changes in metabolites including linoleic acid metabolism, amino acid metabolism, galactose metabolism, and arachidonic acid metabolism. Specifically, the acute CCI group exhibited significant alterations in proline, phosphoric acid, β-hydroxybutyric acid, galactose, creatinine, L-valine, linoleic acid and arachidonic acid. A receiver operating characteristic curve analysis showed that the above 8 metabolites in plasma could be used as the potential biomarkers for the diagnosis of acute TBI. Furthermore, this study is the first time to identify the galactose as a biomarker candidate for acute TBI. This comprehensive metabolic analysis complements target screening for potential diagnostic biomarkers of acute TBI and enhances predictive value for the therapeutic intervention of acute TBI.

## Introduction

Traumatic brain injury (TBI) is the result of direct trauma from an external mechanical force on the brain. TBI is a major cause of mortality and disability worldwide, especially in individuals under the age of 45 years [1]. According to the Centers for Disease Control and Prevention (CDCP), an estimated 1.7 million people in the United States suffer TBI annually [2]. Approximately 5.3 million U.S. residents are living with TBI-induced disabilities, including psychological and long-term cognitive impairments [3]. Increasing evidence indicates that appropriate and timely diagnosis with subsequent intervention in acute TBI can minimize insults and decrease neurological disability [4–6]. Therefore, there is a window of golden opportunity in optimizing the prognosis of TBI patients.

If the molecular mechanism of acute TBI could be deciphered, effective treatments might be developed, and then associated mortality might be reduced. The molecular events including neuroinflammation [7], tauopathy [8], blood-brain barrier (BBB) dysfunction [9] and brain edema [10] characterize the progression of acute TBI. However, the knowledge of the molecular mechanisms of acute TBI is relatively limited due to the multifactorial nature of the acute TBI pathology. Numerus potential molecular mechanisms remain unknown, hindering a comprehensive and effective elucidation of acute TBI. Because of the complicated TBI, single biomarker could not reflect the full spectrum of the response of brain tissue to TBI [11]. Hence, to reveal a global view of the complex and multiple molecular mechanisms of acute TBI is urgently needed [12].

Extensive efforts have focused on the molecular mechanisms underlying acute TBI to identify optimal intervention and therapeutic strategies. Currently, the clinical diagnosis of acute TBI includes brain edema, alterations in cerebral blood flow and metabolic changes [13,14]. Among these changes, the measurement of metabolic alterations in the biofluid is important for the clinical diagnosis. Metabolic progress in the body results in the changes in the concentrations of metabolites. The characterization of the release of these specific metabolites during the metabolic changes could clarify the pathophysiology and the potential therapeutic targets. Several metabolic biomarkers of acute TBI have been proposed, including S100 calcium binding protein B (S100B) [15–18], glial fibrillary acidic protein (GFAP) [19], neuron-specific enolase (NSE) [20,21], sphingolipid (SPL) [22], and medium-chain fatty acids [23]. However, a single biomarker is of limited utility for revealing the mechanism and developing therapeutic strategies in TBI treatment. To break through this limitation, a cluster of biomarker metabolites should be identified to elucidate the characteristics of acute TBI. The biomarkers profile could provide more comprehensive information about metabolic process, in contrast to traditional tests using a single metabolic component during the acute TBI.

Metabolomics, an emerging ‘-omics’ science in systems biology, is a technique that can measure global metabolic profiles related to disease progression [24,25]. Through metabolomics analysis, significant changes associated with disease can be defined as the metabolite changes in the pathological processes of disease. In addition, these changes can be used to identify the potential candidates for biomarkers of disease [26]. Metabolic candidates can be used to decipher the molecular networks of disease via target metabolism pathways [27]. Furthermore, the determination of metabolic profile by metabolomics enable differentiation of the differences between diseased and non-diseased status. Thus, metabolomics is an important strategy for the development of efficient diagnostic methods to improve the diagnosis, prognostication and therapy of disease [28,29]. Metabolomics offers the potential for a holistic approach to clinical diagnosis and treatment as well as an understanding of the pathological mechanisms of complex diseases such as acute TBI [30].

Several metabolomics-based studies of TBI have been published. Orešič M et al. reported a comprehensive metabolic profiling of serum samples from TBI patients and the controls in two independent cohorts [31]. This study showed that two medium-chain fatty acids (decanoic and octanoic acids) and sugar derivatives were strongly associated with the severity of TBI. In addition, Yi L et al. identified several serum metabolite markers and investigated the altered metabolic pathway associated with post-TBI cognitive impairment [32]. Moreover, Glenn TC et al. analyzed the cerebral spinal fluid (CSF) of patients with TBI to create a metabolomic fingerprint for brain injury [33]. What’s more, Ray O et al. evaluated the use of metabolomics for the development of biomarkers for the diagnosis and timing of injury onset in the TBI mouse [34,35]. However, the overall plasma metabolic alterations in acute TBI in rats have not been reported.

This work aimed to investigate the comprehensive metabolomics profile of plasma samples from an experimental controlled cortical impact (CCI) model in rats by gas chromatography/mass (GC/MS) method. We sought to identify metabolite profiles associated acute TBI, further to predict the outcome of acute TBI.

## Materials and methods

### 1. Ethics statement

All procedures conformed to the guidelines for the care and use of laboratory animals established by Central South University. The animal protocols were approved by the Medical Ethics Committee of Xiangya Hospital of Central South University.

### 2. Animal treatment and sample collection

Thirty Sprague-Dawley (SD) rats weighing 200±20 g were provided by the laboratory animal research center of Central South University. Rats were housed in 12h with food and water available *ad libitum* and were acclimated to their environment for at least 1 week prior to any experiment.

After 1 week of adaptive feeding, the 30 SD rats were randomly divided into two groups: the CCI group (n = 15, rats subjected to controlled cortical impact (CCI)) and the sham group (n = 15, rats were shaved only). CCI injury was performed using an electronic controlled pneumatic impact device (TBI0310, precision system & instrumentation LLC, Fairfax Station, VA, USA) according to the previous study [36]. Briefly, the CCI model was established as follows: each rat was initially intraperitoneally anesthetized with 3% pentobarbital sodium (0.45 ml/100g). Then, the head was shaved and the skull was exposed through a midline longitudinal skin incision under a stereotaxic frame and sterile conditions. A 5 mm craniotomy was made over the left parieto-temporal cortex using a portable drill and trephine, with the center of the coordinates 1 mm posterior and 1 mm lateral relative to the bregma before the bone flap was removed. CCI was induced in each rat using the aforementioned device at an impact velocity of 6.0 m/sec and a dwell time of 500 msec. Following impact, the scalp was closed using cyanoacrylate tissue glue. Each of the rats was heated to maintain the body temperature at 37°C throughout the operation. One sham rat died, and 15 CCI rats and 14 sham rats died at the 14th day under anesthesia.

Blood samples were obtained from the rats within the first 24 h after injury. Approximately 150 μl of blood plasma sample was collected with ice and processed within an hour of draw. The plasma was centrifuged and stored at 4°C, and aliquots were prepared and frozen at -80°C until use.

### 3. Modified neurologic score (mNSS) test

In this study, modified neurologic severity score (mNSS) was employed to assess the neurologic deficiency at 1st, 3rd, 7th, and 14th day after TBI. The mNSS was performed including motor, sensory, balance and reflex tests. Once the rat failed to accomplish the task awarded 1 score. The scores ranged from 0 to18, the higher the score, the more severe injury (normal score: 0; maximal damage: 18). We calculated mNSS according to statistical analysis using the SPSS 23.0 (International Business Machines Corporation, Armonk, NY, USA) software. P<0.05 was considered as statistically significant.

### 4. Metabolite determination in rat plasma using GC/MS

Each 100 μl plasma was mixed with 300 μl of methanol as extraction. 50 μl of heptadecanoic acid (dissolved in 1 mg/ml of methanol) was added as internal standard. After vortexed for 60 s and centrifuged (10 min, 16000 rpm, 4°C), the supernatant was transferred to a 5 ml glass centrifugation tube, and then evaporated to dryness under nitrogen. 50 μl of methoxyamine/pyridine (20 mg/ml) was added to the tube for methoximation for 1 h at 70°C. Afterward, as derivatization agent, 100 μl of N,O-Bis (trimethylsilyl) trifluoroacetamide (BSTFA) was added to the residue, and then incubated for 1 h at 70°C. Finally, the sample was injected into GC/MS system for analysis.

The sample solution was injected into a Shimadzu GC-2010 gas chromatography instrument coupled to a Shimadzu QP2010 mass spectrometer (Shimadzu, Kyoto, Japan) for GC/MS analysis according to the published guidelines [36]. The mass spectrometry conditions were maintained as follows: ionization voltage, 70 eV; ion source temperature, 200°C; interface temperature, 250°C; full-scan mode in the 35–800 amu mass range with 0.2 s scan velocity; and detector voltage, 0.96 kV. After a 4-min solvent delay time maintained at 70°C, the oven temperature was increased in increments of 8°C/min from 70°C to 300°C and held for 3 min. The injection temperature was set at 280°C, with unbolting of the septum purge at a flow rate of 3 ml/min. Helium was used as the carrier gas at a flow rate of 1 ml/min.

Raw spectrometric data including retention time (RT), chromatographic peak intensities and the integrated mass spectra of each plasma sample, were applied for the analysis. In addition, available commercial standards were used for confirmation. Only metabolic features with a relative standard deviation (RSD) for the relative peak areas of <30% in quality control (QC) samples were retained for the subsequent data analysis. The peak areas of metabolites were normalized to the internal standard to obtain the semi-quantitative level of metabolites for further statistical analysis. A data matrix, in which the rows represented the samples and the columns corresponded to the peak area ratios relative to the internal standard in the same chromatogram, was generated by extracting the peak areas using our custom scripts.

To identify and quantify the individual metabolites from the GC/MS spectra, individual peak retention time and the online mass spectrometry (MS) spectra were compared with the authentic standard. Metabolites identification was unambiguously identified and validated by the reference standards. The other components were identified in chromatograms based on their electron bomb ionization (EI) MS data available in the National Institute of Standards and Technology (NIST) library. We comprehensively searched the NIST/EPA/NIH Mass Spectra Library (NIST05) and the characteristic ions for similar electron impact spectra corresponding to the structures of the peaks-of-interest using NIST Mass Spectral Search Program Version 2.0. After the peak areas were extracted to generate a 3-dimensional data matrix, the matrix with mass spectra was processed, including data extraction, peak matching, retention time adjustment, visualization and normalization for multivariate statistical analysis.

### 5. Data processing and statistical analysis

For each metabolite identified by GC/MS in this study, detailed analysis of the statistical significance was performed using the nonparametric Mann–Whitney U test [37]. A value of P<0.05 indicated a statistically significant difference. Statistical analysis was conducted using SPSS 23.0 (International Business Machines Corporation, Armonk, NY, USA) software. Missing values for each metabolite were replaced with the mean value. We then performed pareto-scaling and power transformation for all samples after principal component analysis (PCA).

PCA of the data set was performed to visualize the profiles of plasma metabolites between the acute CCI and sham groups in this study. Then, the predicted probabilities of metabotypes among the CCI and sham rats were analyzed using partial least squares-discriminant analysis (PLSDA). A prediction model was constructed as a valid model for biomarkers differentially expressed between the acute TBI and sham groups. The goodness of fit (R2Y(cum) and Q2Y(cum)) of this model and permutation tests were performed to validate the accuracy and assess the risk of the supervised PLSDA model with at least 100 iterations of permutation for each test. Meanwhile, S-plot is widely used in MS-based metabolomics studies for identification of significant biomarkers combined with orthogonal partial least square discriminant analysis (OPLSDA) [38,39]. The potential biomarker metabolites in CCI rats were further identified based on variable importance in the projection (VIP) values calculated from the OPLSDA model with a threshold of 1.2 and S-plot. VIP metabolites with P values of <0.05 with hierarchical S-plot following analysis of variance of the cross-validated residuals (CV-ANOVA) across the two subject groups were considered statistically significant candidate metabolic biomarkers in this study. All samples were exported to the SIMPCA-P 13.0 platform (Umetrics AB, Umea, Sweden) for PCA, PLSDA and OPLSDA.

Additionally, fold-change analysis was performed by dividing the relative abundances of the corresponding metabolite. Q values were calculated using the false discovery rate (FDR) for multiple testing corrections [40]. In our study, biomarkers that showed a significant fold-change of ≥1.2 and an FDR value of ≤0.05 in acute TBI vs. Sham was submitted to identification. Finally, the area under the receiver operating characteristic (ROC) curve was computed to evaluate the overall performance of the biomarkers in acute TBI for detecting difference factors [41]. An area under ROC curve (AUC) of 1 is considered to have perfect discriminatory power, whereas a value of 0.5 suggests that the discriminatory power is no better than chance. All FDR control and ROC curves were constructed using the freely available software SPSS 23.0 (International Business Machines Corporation, Armonk, NY, USA) with the corresponding packages.

### 6. Metabolomics analysis

To explore complex associations between multiple parameters collected from samples, heatmaps derived from the normalized data were obtained using the correlation-based cluster program and visualized using Metaboanalyst 3.0 (http://www.metaboanalyst.ca/). In this study, a red/green color scheme in the heatmaps represents the level of fold change of a metabolite relative to the median concentration between the rats in the acute CCI and Sham groups, respectively. To represent common and specific features of metabolites, a Venn diagram was used to classify significantly changed metabolites. To illustrate the links in the potential biomarker metabolic pathways, we used pathophysiology, biochemistry, physiology and rat metabolome databases to provide quantitative and metabolic information on metabolites associated acute TBI. Analysis of the significantly changed metabolites and pathway impact analysis of the metabolites were performed using Metaboanalyst 3.0 (http://www.metaboanalyst.ca/) and KEGG (Kyoto Encyclopedia of Genes and Genomes).

## Results

### 1. mNSS results

**Fig 1:**
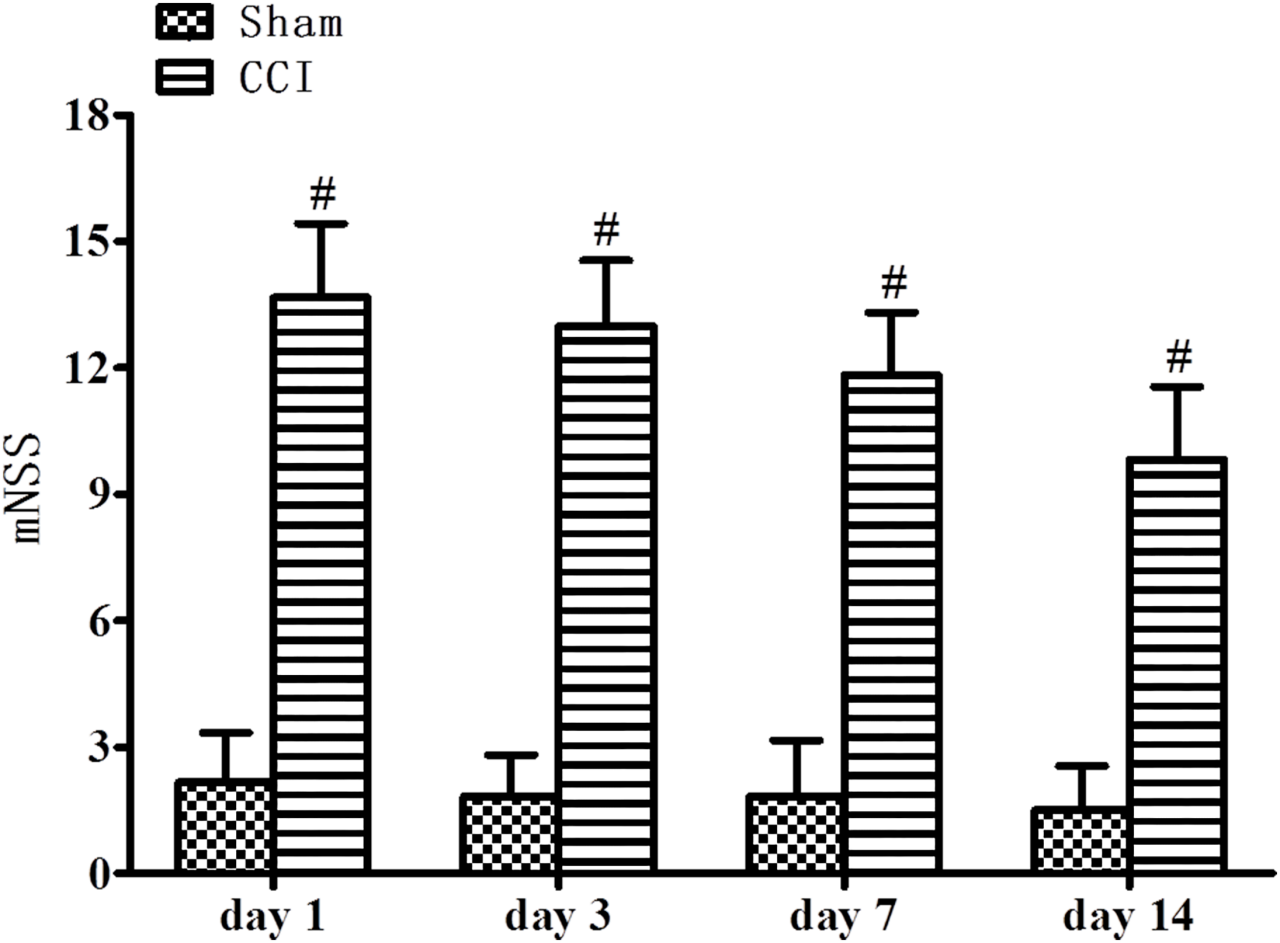
mNSS tests: TBI-induced injury groups noteworthy high scores when compared with sham group (n = 6). #p<0.01 vs. Sham group.

As shown in Fig 1, Neurological function was evaluated by mNSS on 1st, 3rd, 7th, and 14th day after TBI. All CCI rats exhibit marked higher scores than the sham group (p<0.01). Our results demonstrated that CCI induced significant performance outcome compared with the sham group according to the mNSS tests.

### 2. Univariate data analysis of 45 GC/MS-based plasma metabolites of acute TBI

**Fig 2:**
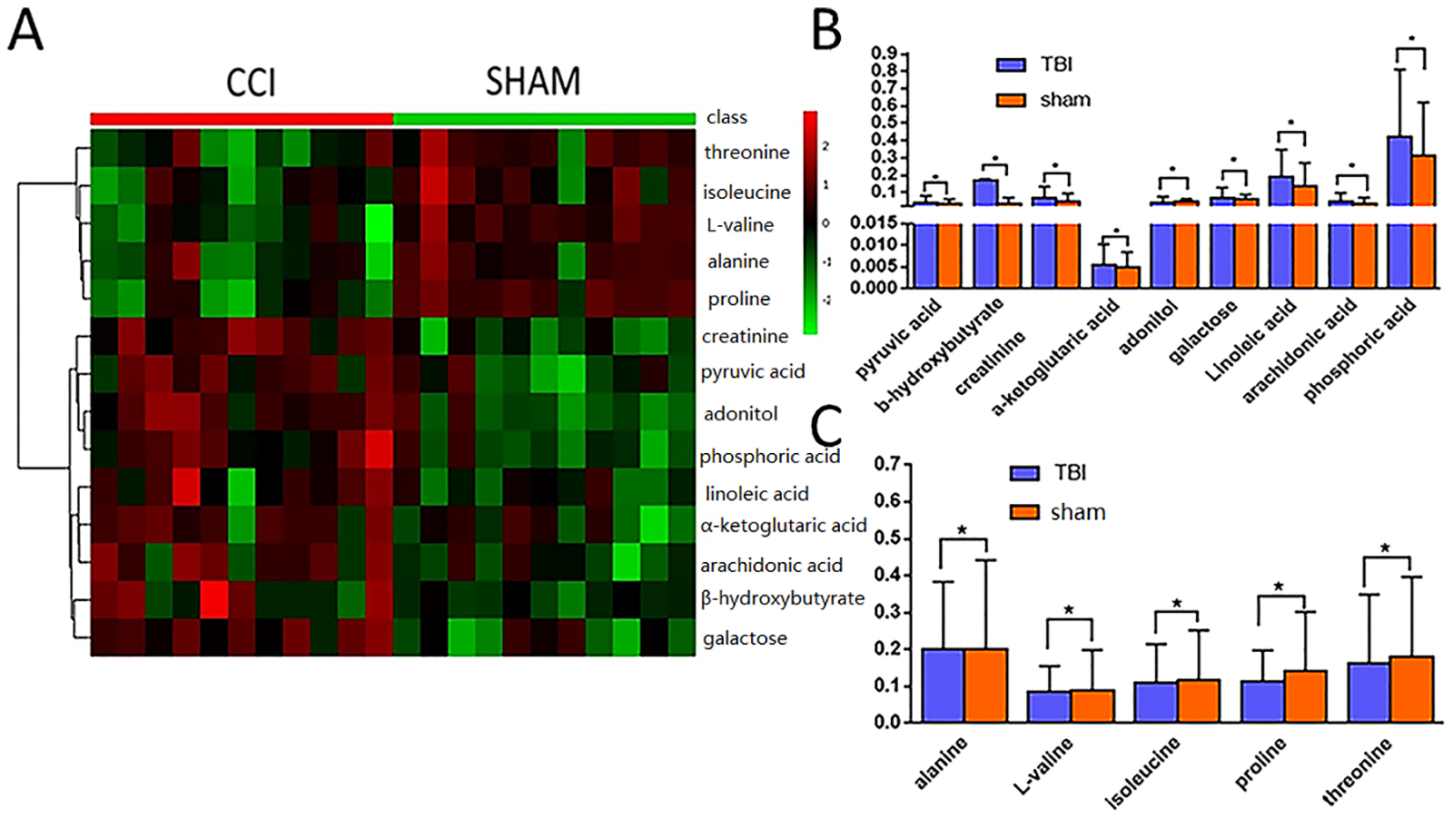
Performances of statistically significant metabolites in acute TBI. (A) Hierarchically clustered heatmap of 14 statistically significant metabolites. Sham-operated (green) and TBI-injured (red) animals clustered into separate clusters. From top down, the relative concentrations of threonine, alanine, isoleucine, L–valine, and proline increased successively, and the relative concentrations of β-hydroxybutyrate, adonitol, creatinine, arachidonic acid, α-ketoglutaric acid, pyruvic acid, galactose, phosphoric acid, and linoleic acid successively decreased. (B) Bar graphs of statistically significant metabolites: the values in the acute TBI group were greater than those in the sham group. (C) Bar graphs of statistically significant metabolites: the values in the acute TBI group were lower than those in the sham group.

A GC/MS-based approach was employed to determine plasma metabolic changes in acute TBI. As shown in Table 1, 45 metabolites were identified. The above metabolites belonged to metabolic processes related to amino acids, organic acids, lipids, carbohydrates, urea and energy. Among these metabolites, a total of 14 metabolites in plasma were significantly altered in acute CCI rats compared with the sham group (* in Table 1). Five metabolites increased strikingly in acute CCI rats compared with the sham group, whereas the levels of 9 metabolites decreased markedly. Using the nonparametric Mann–Whitney U test (p<0.05), heatmap visualization of the clustering of metabolite profiles based on 14 statistically significant metabolites demonstrated a statistically significant correlation between all samples originating from the left (the acute CCI group) to the right (the sham group) (Fig 2A). Metabolites following acute TBI that contributed significantly included successive decreases in the relative values of β-hydroxybutyric acid, adonitol, creatinine, arachidonic acid, α-ketoglutaric acid, pyruvic acid, galactose, phosphoric acid, and linoleic acid for up regulation (Fig 2B) and successive increases in the relative values of threonine, alanine, isoleucine, L-valine, and proline for down regulation (Fig 2C).

**Table 1:** Qualitative and quantitative analysis of the metabolic profiles of the acute TBI and sham groups; the data of 45 metabolites are presented as the mean±SD.

No.	Metabolites	First 24 hours		KEGG	HMDB
		TBI (n = 15)	Sham (n = 14)		
1	Pyruvic acid*	0.085±0.019	0.065±0.014	C00022	HMDB00243
2	Lactic acid	5.005±0.852	4.353±0.357	C00186	HMDB00190
3	Alanine*	0.411±0.098	0.479±0.063	C01401	HETPA0179
4	Glycine	0.343±0.302	0.248±0.030	C00037	HMDB00123
5	Methylmalonic acid	0.061±0.011	0.057±0.008	C02170	HMDB00202
6	N-acetylglycine	0.077±0.010	0.075±0.008	-	HMDB00532
7	β-hydroxybutyrate*	0.178±0.168	0.075±0.016	C01089	HMDB00357
8	L—valine*	0.166±0.043	0.216±0.028	C00183	HMDB00883
9	Urea	2.986±0.678	2.942±0.705	C00086	HMDB00294
10	Isoleucine*	0.231±0.048	0.273±0.038	C16434	HMDB33923
11	Proline*	0.211±0.065	0.327±0.049	C00148	HMDB00162
12	Succinic acid	0.027±0.005	0.024±0.005	C00042	HMDB00254
13	Glyceric acid	0.045±0.016	0.039±0.015	C00258	HMDB00139
14	Fumaric acid	0.013±0.007	0.012±0.006	C00122	HMDB00134
15	Serine	0.284±0.051	0.301±0.044	C00065	HMDB00187
16	Threonine*	0.378±0.056	0.429±0.058	C00188	HMDB00167
17	Malic acid	0.029±0.010	0.027±0.007	C00711	HMDB00744
18	Focal glutamate	0.598±0.100	0.498±0.182	C01879	HMDB00267
19	4—hydroxyproline	0.092±0.020	0.093±0.013	C01157	HMDB00725
20	2,3,4-hydroxybutyrate	0.051±0.022	0.040±0.016	C01620	HMDB00943
21	Creatinine *	0.142±0.034	0.101±0.019	C00791	HMDB00562
22	α-Ketoglutaric acid *	0.011±0.003	0.009±0.003	C00026	HMDB00208
23	Ornithine	0.050±0.014	0.062±0.026	C00077	HMDB00214
24	Glutamate	0.096±0.028	0.079±0.016	C00217	HMDB03339
25	Phenylalanine	0.077±0.011	0.084±0.014	C00079	HMDB00159
26	Aspartic acid	0.031±0.018	0.032±0.013	C00402	HMDB06483
27	Adonitol*	0.080±0.018	0.058±0.018	C00474	HMDB00508
28	Lysine	0.062±0.036	0.066±0.041	C00047	HMDB00182
29	Glutamine	0.028±0.010	0.039±0.030	C00064	HMDB00641
30	Citric acid	0.068±0.024	0.071±0.021	C00158	HMDB00094
31	1, 5-anhydroglucitol	0.125±0.023	0.127±0.037	C07326	HMDB02717
32	Fructose I	0.043±0.014	0.054±0.030	C00095	HMDB00660
33	Galactose*	0.137±0.032	0.092±0.044	C00124	HMDB00143
34	Glucose I	7.107±1.869	7.385±1.456	C00031	HMDB00122
35	L-tyrosine	0.078±0.017	0.092±0.022	C00082	HMDB00158
36	Palmitic acid	0.577±0.159	0.473±0.089	C00249	HMDB00220
37	Tryptophan	0.008±0.006	0.012±0.006	C00525	HMDB13609
38	Inositol	0.157±0.037	0.133±0.020	C00137	HMDB00211
39	Margaric acid	0.013±0.003	0.011±0.003	-	HMDB02259
40	Linoleic acid*	0.371±0.097	0.292±0.056	C01595	HMDB00673
41	Oleic acid	0.349±0.091	0.286±0.041	C00712	HMDB00207
42	Stearic acid	0.301±0.065	0.280±0.072	C01530	HMDB00827
43	Arachidonic acid*	0.104±0.023	0.074±0.017	C00219	HMDB01043
44	Cholesterol	0.408±0.162	0.332±0.083	C00187	HMDB00067
45	Phosphoric acid*	0.872±0.195	0.669±0.130	C00009	HMDB01429

* A p value of <0.05 in the Mann-Whitney U test between the acute TBI and sham groups was considered statistically significant.

### 3. Metabolic pattern analysis of acute TBI

**Fig 3:**
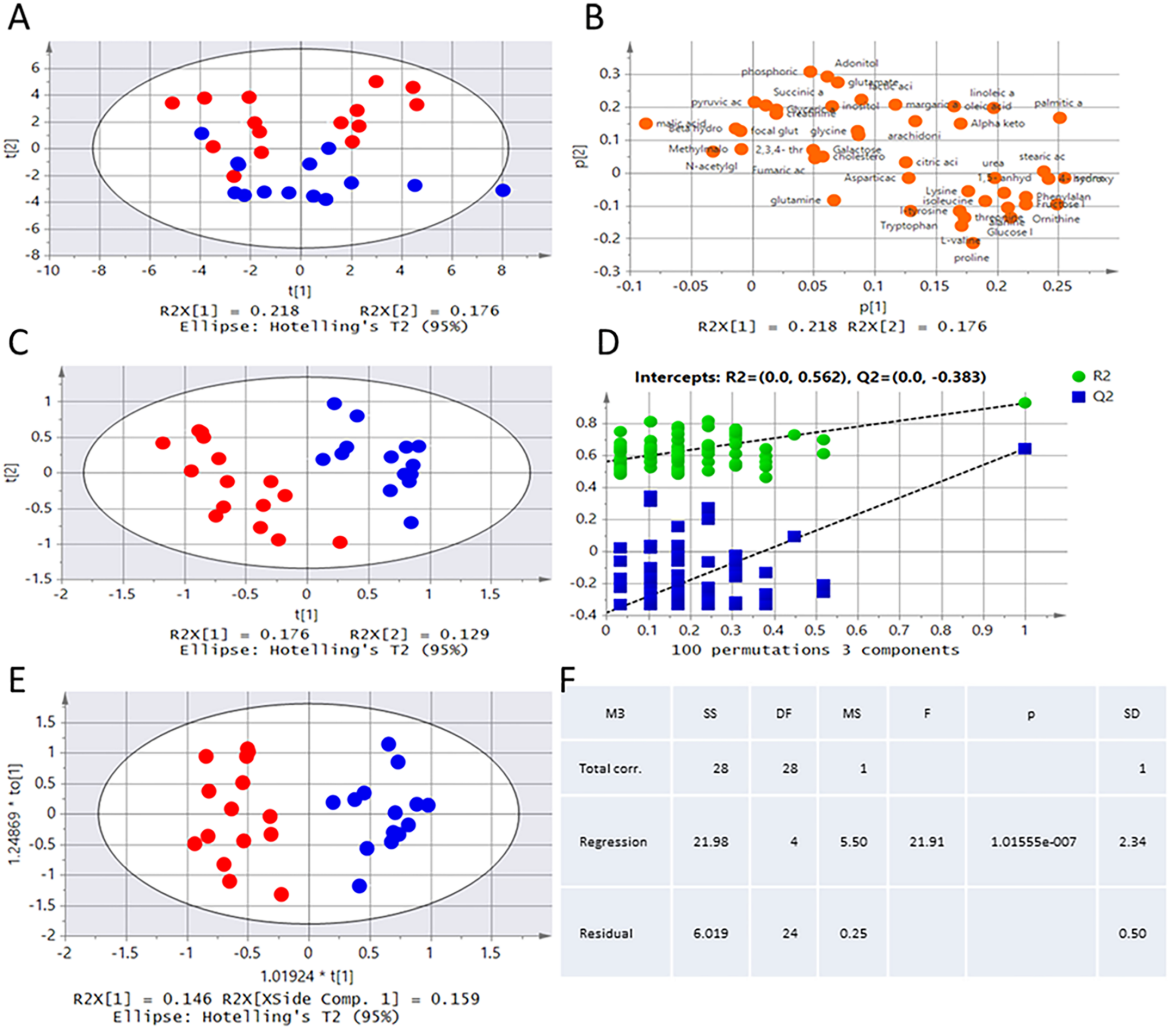
Multivariate presentation of the acute TBI (red) and sham groups (blue) by multivariate data analysis. (A) PCA of metabolites in the acute CCI and sham rat groups. (B) PCA loading of metabolite data. The metabolites had a major influence on the separation. (C) PLSDA of metabolites in the acute CCI and sham rat groups. (D) Validation plots of the PLSDA models acquired through 100 permutation tests for TBI vs health. (E) OPLSDA of metabolites in the acute CCI and sham rat groups. (F) Validation of the OPLSDA from an ANOVA of the cross-validated predictive residuals (CV-ANOVA). All data analysis was performed to indicate significant detectable differences between the acute CCI and sham rat groups.

An unsupervised PCA model was used to classify acute TBI subjects in the animal group to support utility in diagnostic model building. The obtained PCA model showed that the first 2 principal components explained 39.4% of the variance and effectively separated the samples into two clusters (Fig 3A). The metabolites were represented by dots to construct a PCA loading plot, and the dots were considered to contribute more to the model classification of acute TBI in the direction of the separation of PCA (Fig 3B). To make this distinction more evident, PLSDA with the pareto-scaled data set and power transform was performed using the first two latent variables (Fig 3C). In the PLSDA model, R2Y (cum) = 0.927, and Q2Y (cum) = 0.644, indicating that the model had perfect fitting and reliable predictive ability. To validate the validity of the PLSDA model, randomization (n = 100) in permutation tests was performed. As shown in Fig 3D, R2 = 0.562, and Q2 = -0.383, indicating that the PLSDA model is reliable and optimal at classifying and discriminating between the sham and acute TBI groups. When the metabolic discrimination models established by PLSDA were verified, the highest differentiation of the acute TBI and Sham groups found by OPLSDA was used to indicate significant detectable differences (Fig 3E). The fit of the models was assessed based on R2 (cum) = 0.897 and the predictive capability parameter Q2 (cum) = 0.785. The significance of the OPLSDA models was estimated based on P = 1.01555e-007, i.e. P<0.01, from ANOVA of the cross-validated predictive residuals (CV-ANOVA).

### 4. VIP plasma metabolite-based identification of acute TBI and multivariate data verification

**Fig 4:**
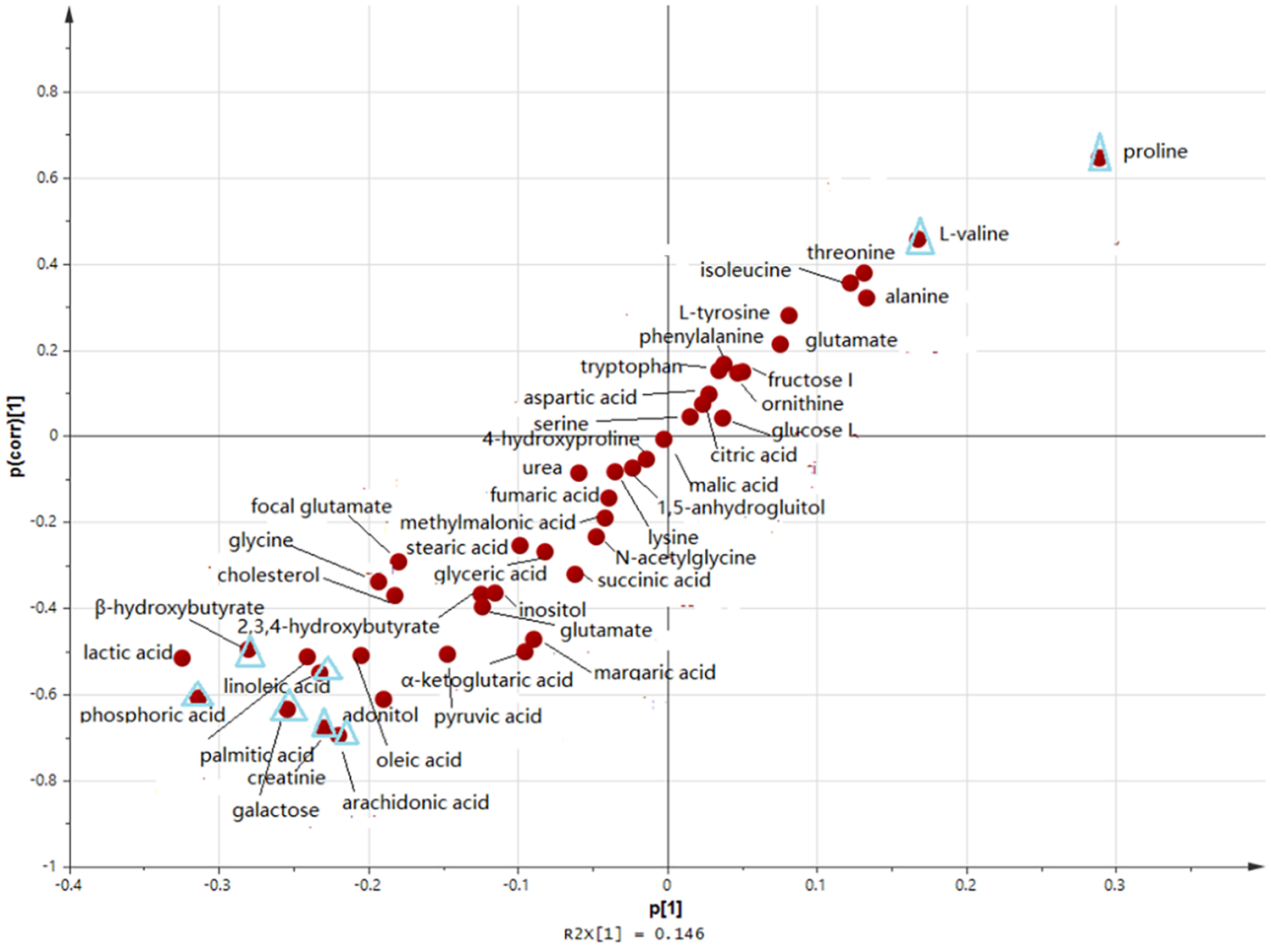
OPLSDA S-plot of the acute CCI vs sham groups.

**Fig 5:**
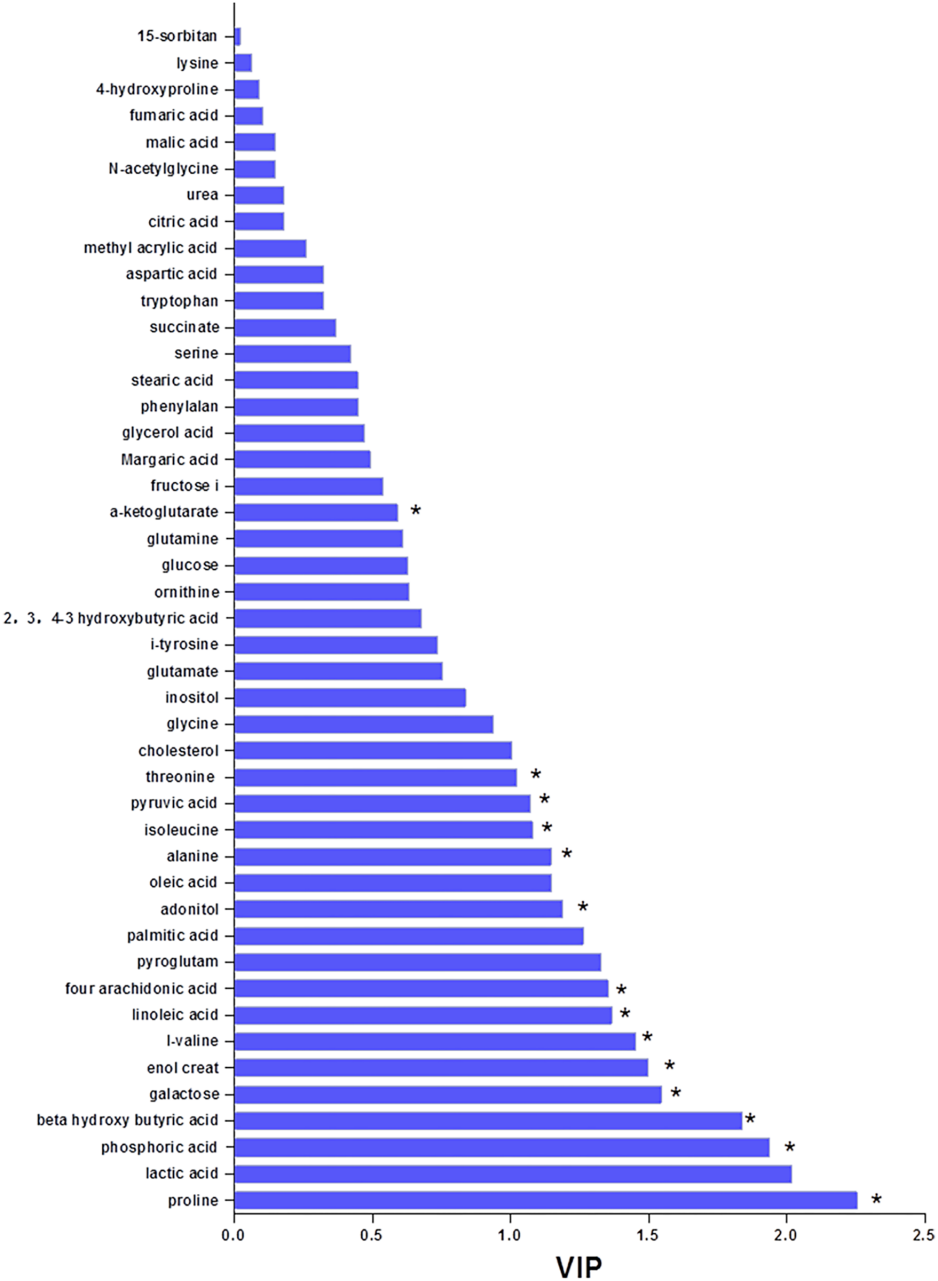
Variable importance in the projection (VIP) value plots of each metabolite discriminating the acute CCI and sham rat groups. The VIP plot displays the top 8 metabolites (proline, phosphoric acid, β-hydroxybutyric acid, galactose, creatinine, L-valine, linoleic acid, and arachidonic acid) identified by OPLSDA. The VIP value is a weighted sum of squares of the OPLSDA loadings taking into account the amount of explained variable in each dimension. * A P value of <0.05 in the Mann-Whitney U test between the TBI and sham groups was considered statistically significant.

Top metabolites were selected from OPLSDA using the S-plot of 45 metabolites identified (Fig 4). Meanwhile, the VIP values of the 45 metabolites were calculated based on the OPLSDA model, and VIP metabolites with VIP value>1.2 were selected from Fig 5. Of the top 11 metabolites identified by VIP scores and S-plot, proline, lactic acid, phosphoric acid, β-hydroxybutyric acid, galactose, creatinine, L-valine, linoleic acid, arachidonic acid, pyroglutamate and palmitic acid were identified as metabolite variables that significantly contributed to the class separation of the acute TBI and sham groups based on significantly altered plasma features (Fig 5). Taken together, the univariate and multivariate statistical analysis demonstrated that 8 VIP metabolites (proline, phosphoric acid, β-hydroxybutyric acid, galactose, creatinine, L-valine, linoleic acid, and arachidonic acid) of the 11 VIP metabolites were the potential candidates for a diagnostic biomarker panel in rats after induction of acute TBI (Table 2).

**Table 2:** List of differential VIP metabolites derived from the OPLSDA model of GC-MS analysis between the acute TBI and sham groups.

Metabolites	TBI vs sham			
	VIP^a^	FC^b^	p-VALUE*	Q-VALUE
1.Linoleic acid	1.3659	1.27	0.018	0.023
2. β-hydroxybutyric acid	1.8385	2.37	0.016	0.022
3.Phosphoric acid	1.9392	1.3	0.006	0.009
4.Galactose	1.5458	1.49	0.002	0.007
5.L-valine	1.4528	0.77	0.002	0.006
6.Arachidonic acid	1.3527	1.41	0.001	0.005
7.Proline	2.2548	0.65	0.00	0.00
8.Creatinine	1.4993	1.41	0.00	0.00

^a^, variable importance in the projection;

^b^, fold change;

* values were calculated using the nonparametric test.

**Fig 6:**
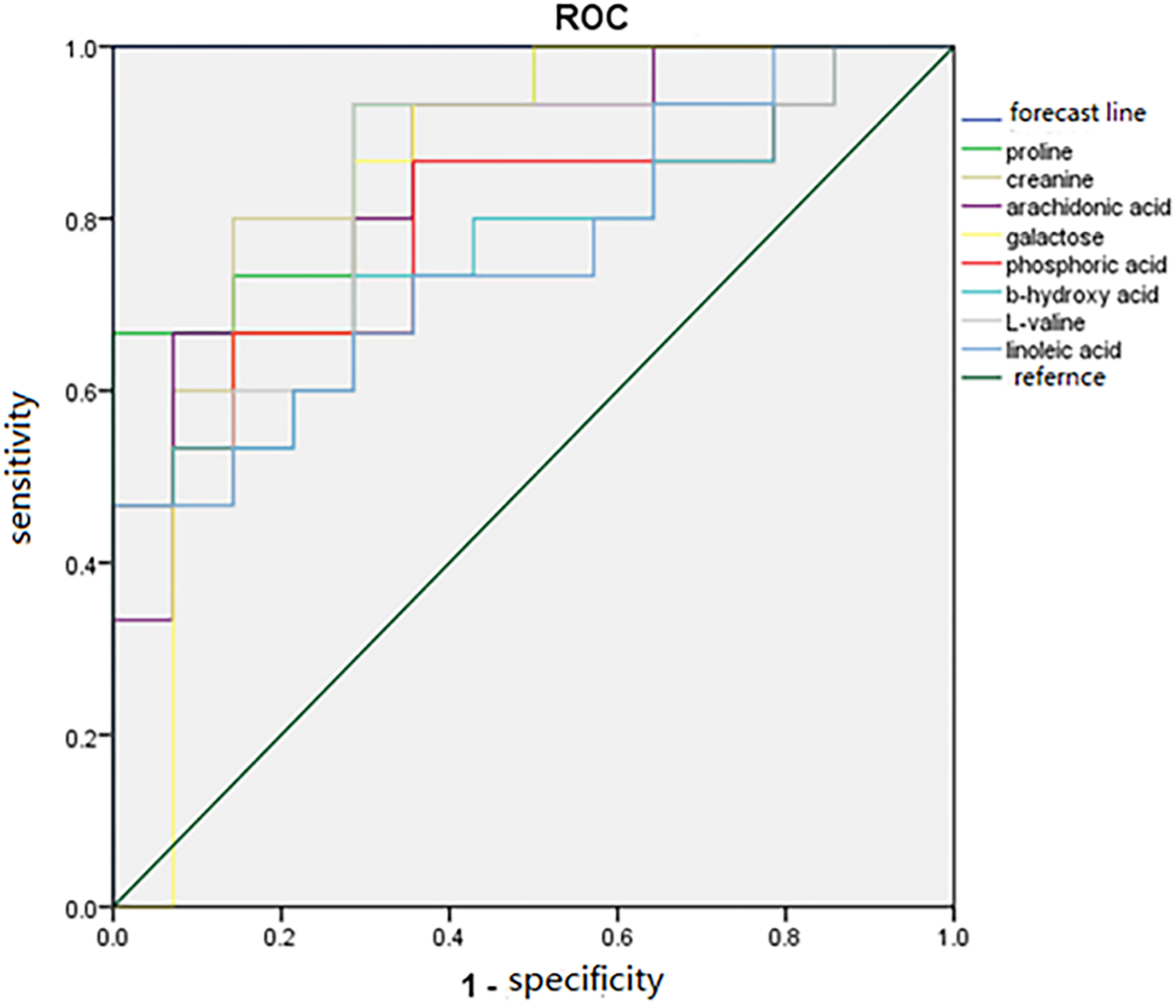
Comparison of the area under the ROC curve (AUC) of VIP based on OPLSDA: Proline (AUC 0.90), creatinine (AUC 0.88), arachidonic acid (AUC 0.85), galactose (AUC 0.83), phosphoric acid (AUC 0.80), β-hydroxybutyric acid (AUC 0.76), L-valine (AUC 0.83), and linoleic acid (AUC 0.76). All AUCs were above 0.5, indicating that acute TBI can be identified based on these 8 predictors.

To confirm the identification of the 8 VIP metabolites, relative abundance and multiple testing corrections were performed. Table 2 presents the list of differential VIP metabolites with the fold change (FC) and Q values between TBI-treated and untreated rats. In Table 2, FC >1.2 with a value >1.0 indicates a higher value in the acute TBI affected rats compared to unaffected rats, while a value <1.0 indicates lower value compared to the sham group. β-Hydroxybutyric acid increased most dramatically after acute TBI, whereas the increase in linoleic acid was the smallest. Moreover, FDR analysis of the 8 VIP metabolites of interest ranked the metabolites based on their adjusted p value, and all Q values were less than 0.05, confirming valid conditions for multiple variable testing in this study. Furthermore, ROC analysis with the true-positive rate (sensitivity) and false-positive rate (1-specificity) of the 8 VIP candidates was used to evaluate the possibility of using these markers for diagnosing acute TBI (Fig 6). These 8 metabolic candidates showed significant changes between the sham and acute TBI samples with good ROC performance (all AUC>76%) as obtained by calculating the value of each VIP metabolite in acute TBI cases. Among these, proline, with AUC of 90%, had the highest diagnostic accuracy for acute TBI in this study. Following proline, the strength of the association of each parameter with acute TBI decreased in the order creatinine (AUC 0.88), arachidonic acid (AUC 0.85), galactose (AUC 0.83), L-valine (AUC 0.83), phosphoric acid (AUC 0.80), β-hydroxybutyric acid (AUC 0.76) and linoleic acid (AUC 0.76), according to the AUCs in ROCs. Regression analysis also showed that the best optimal diagnostic model could be achieved using a combination of the 8 identified VIP metabolites (Fig 6). Taken together, these results indicated that these 8 discriminators were not only strong predictors in differentiating between TBIs and non-TBIs, but also may have potential clinical utility in supporting an acute TBI diagnosis.

### 5. Eight significant metabolites of acute TBI and their 4 correlation pathways

**Fig 7:**
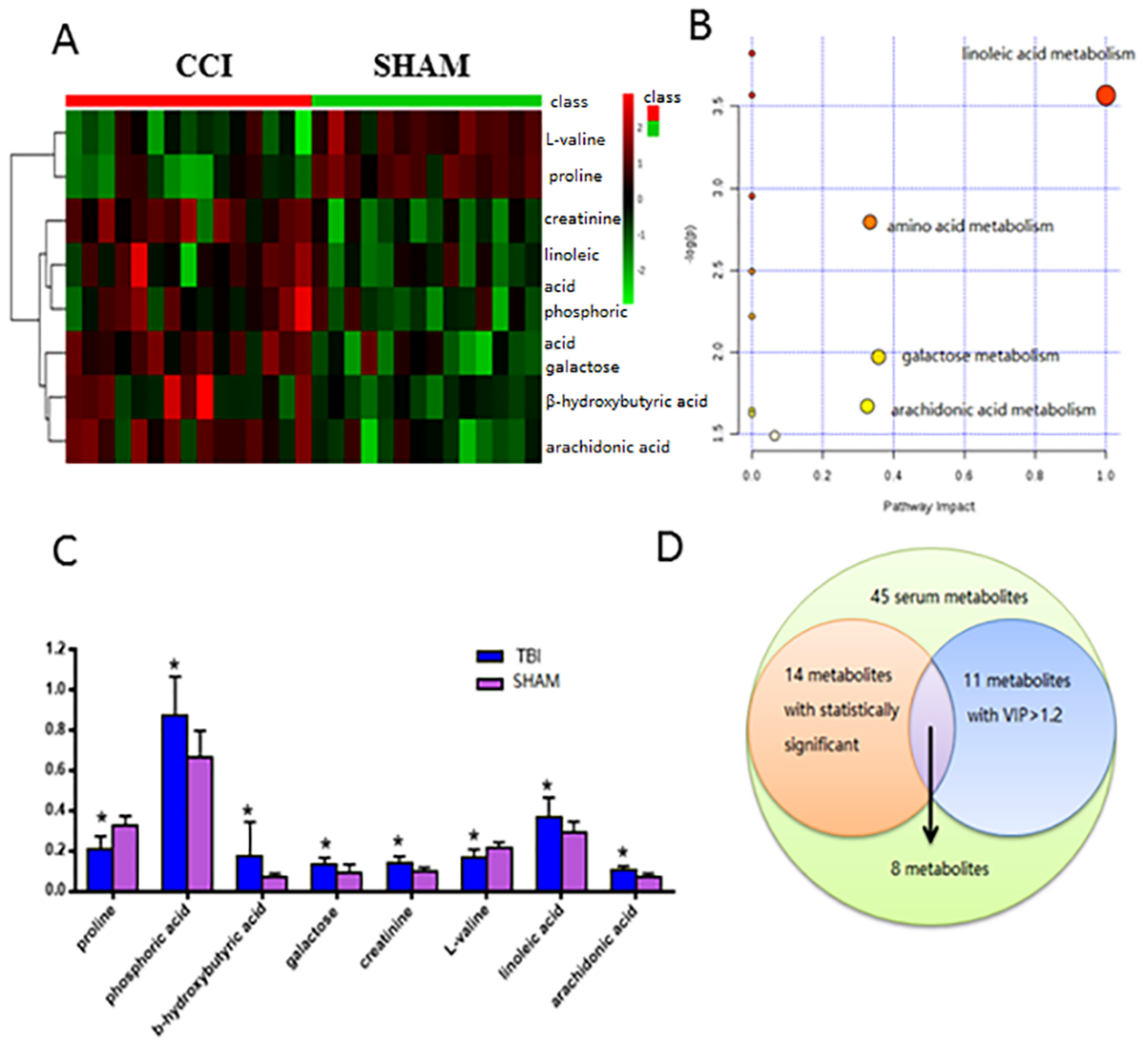
Visualization of the pool of potential biomarkers. (A) Hierarchically clustered heatmap of 8 VIP metabolites with statistical significance. Sham-operated (green) and acute TBI-injured (red) animals clustered into separate clusters. (B) Summary of the analysis of altered metabolic pathways. Four metabolic pathways of importance were disturbed in plasma samples from the acute TBI group: linoleic acid metabolism; galactose metabolism; amino acid metabolism; and arachidonic acid metabolism. (C) Bar graphs of 8 VIP metabolites with statistical significance: the values of the 6 metabolites in the sham group were lower than those in the TBI group. (D) Venn diagram depicting the proportions of plasma metabolites, VIP metabolites and statistically significant metabolites selected in acute TBIs based on metabolomics.

Metabolic biomarker groups were examined and identified using hierarchical cluster analysis. The results are visualized as a heatmap in Fig 7A. Red colors indicate high values for phosphoric acid, β-hydroxybutyric acid, galactose, creatinine, linoleic acid, and arachidonic acid in acute TBI, and green colors indicate lower concentrations of proline and L-valine following acute TBI.

Following hierarchical cluster analysis, metabolic pathway analysis was performed to identify the pathways affected by these eight candidates. All annotated metabolites were mapped to terms in the KEGG database to search for significantly enriched metabolites in metabolic or signal transduction pathways. A total of 4 important metabolic pathways were identified as disturbed in acute TBI, including linoleic acid metabolism, amino acid metabolism, galactose metabolism and arachidonic acid metabolism (Fig 7B). This metabolomics view of all matched pathways involved in acute TBI was performed based on the p values of enrichment analysis and pathway impact values from pathway topology analysis. Linoleic acid metabolism, amino acid metabolism, galactose metabolism, and arachidonic acid metabolism were disturbed successively in CCI rats.

The bar plots of the comparison of the values of these 8 potential metabolic biomarkers between the acute TBI and sham groups are shown in Fig 7C. Only two reduced metabolites (proline and L-valine) were used as the determinants of acute TBI compared to the sham group. By contrast, the remaining six metabolites, phosphoric acid, β-hydroxybutyric acid, galactose, creatinine, linoleic acid, and arachidonic acid, were elevated during acute TBI. Moreover, a Venn diagram was constructed to show the numbers of statistically significant metabolites or VIPs in the acute TBI vs Sham groups (Fig 7D). 8 of 14 metabolites with statistical significance were found to be involved in the induction of 11 VIP metabolite biomarkers in acute TBI (Fig 7D).

## Discussion

In this study, we used a non-target metabolomics approach to perform a comprehensive analysis of metabolic alterations in the plasma of acute CCI rats based on a GC/MS method. It is the primarily study to identify plasma potential biomarkers and unravel the metabolic mechanisms of acute TBI.

Our data from the metabolic phenotypes revealed a good description of disease-specific patterns of acute TBI in plasma metabolomics. In addition, 45 metabolites were significantly altered in the plasma of CCI rats compared with the sham group during the acute phase based on GC/MS measurements. 8 metabolites (proline, phosphoric acid, β-hydroxybutyric acid, galactose, creatinine, L-valine, linoleic acid and arachidonic acid) in plasma with statistical significance were selected as significant metabolic candidates by OPLSDA combining with multivariate and univariate statistical analysis. Furthermore, the above metabolites were strongly relevant to metabolic pathways including linoleic acid metabolism, amino acid metabolism, galactose metabolism and arachidonic acid metabolism using KEGG database and Metaboanalyst tool. These results can be used to identify the identified changes in the metabolic profiles that are mainly associated with the pathophysiological mechanism of acute TBI. These changes suggested that acute TBI may involve in universal metabolic disturbances with complicated molecular interactions and regulations occurring in the development of TBI. These biological approaches successfully contributed to the excellent predictive values for distinguishing acute TBI type from non-TBI.

Previous studies have shown that amino acids (proline, L-valine), phosphoric acid, lipids fatty acids (linoleic acid, arachidonic acid), creatinine and carboxylic acid (β-hydroxybutyric acid) are relevant to TBI [42–48]. As shown in this study, the plasma of proline and L-valine were significantly lower, whereas the phosphoric acid, β-hydroxybutyric acid, creatinine, linoleic acid and arachidonic acid were significantly higher in acute CCI rats compared with the sham group. These results are consistent with previous reports [49–51]. These 7 metabolite components provide a prognostic value for the pathogenesis of acute TBI. Of these markers, proline was recognized not only as a promising biomarker of post-traumatic neurological deficit, but also as a sign of brain damage severity for the monitoring of TBI patients [52]. Phosphoric acid serves as a new type of hybrid material functionalized mesoporous organo-silica nanoparticle [53] and as a catalyst applied to food waste by the hydrothermal method [54]. β-hydroxybutyric acid (BHBA) is thought to inhibit hormone regulation and trigger the BHBA-mediated mechanism regulated growth hormone-releasing hormone (GHRH) synthesis and secretion [55]. Meanwhile, the effects of beta-hydroxybutyrate (BHB) on brain vascular permeability show that BHB leads to BBB disruption in animals [56]. Quantitative assessment of creatinine supports the clinical diagnosis of renal impairment [57], and provides information on the renal function [58]. Furthermore, serum creatinine is associated with all-cause dementia [59]. L-Valine, L-leucine, L-isoleucine, L-phenylalanine, and L-tyrosine are considered as proposed diagnostic indicators for the early detection and diagnosis of type 2 diabetes [60]. Linoleic acid causes red blood cells injury and hemoglobin damage via oxidative mechanism [61] and is a potential biomarker for advanced non-small cell lung cancer [62]. Arachidonic acid is the major polyunsaturated fatty acid and its involvement in Alzheimer’s disease mechanism [63]. Furthermore, arachidonic acid involves diapedesis in the initiating steps of prostaglandins and leukotriene and induces inflammatory cellular infiltration [64]. These raise the possibility of therapy to improve the prognosis of acute TBI. Nevertheless, the 7 metabolites were assessed in previous study by offering each physiological and pathological understanding of TBI as a diagnostic tool [65–74]. By using our metabolomics platform in this study, these 7 plasma metabolites (statistically important with VIP >1.2) were assessed. Hence, it is the plasma metabolome that was indicative of acute TBI-associated global changes in metabolism rather than that base on the single physiological and pathological mechanism of TBI. Our GC/MS plasma metabolomics shows great promise for determining the metabolic signatures from biofluids as a biomarker discovery tool. Further study of a global and associated physiological and pathological process for acute TBI may be facilitated based on the dynamic metabolic alterations and their interactions of these candidates that occur in pathological process of acute TBI.

In the present study, galactose metabolism is firstly observed significant pathologically changes as a candidate in rats with acute TBI. The plasma metabolic profiles that we measured in this study indicate that acute CCI rats had higher galactose level by using our metabolic platform including OPLSDA, VIP and multivariate analysis. The higher plasma galactose level in acute CCI rats indicates that galactose could be viewed as an early marker for TBI. It is shown that the metabolic platform can allow a novel potential plasma biomarker whose function has not been elucidated for acute TBI. This suggests that plasma metabolomics may be a novel approach for determining comprehensive characterization of complicated disease. Moreover, Galactose has been reported previously to influence the establishment of infection [75–77], and to contribute to the pathophysiology of classical galactosemia [78]. D-galactose (DG) has been examined in aging mice [79]. The protective effect of DG improved cognitive impairment and neurodegenerative disease [80,81]. Galactose was also reported that it was significantly higher in TBI patients without cognitive impairment as well as AD animal model based on metabolic approach [82]. Since the galactose plasma metabolite associated with acute CCI rat in this study was also changed in AD and in post-TBI cognitive impairment, we could speculate that during acute TBI, AD and cognitive deficits may share similar pathophysiological changes at the metabolic level. This hypothesis can further help to elucidate the extent of disease processes of acute TBI related infection, cognition and Alzheimer's disease. Although more researches on galactose and acute TBI should be targeted validated, this process appear to open the possible way of the pathological research of acute TBI.

It was also worthy to note that other 37 metabolites of these 45 metabolites were not identified to be metabolic candidates of acute TBI in this study. However, most of these metabolites have been reported in clarifying pathophysiological characters of TBI with highly contribution. For instance, myo-inositol combined with the organic osmolytes taurine can discriminate the different time groups of brain edema which response to follow-up the progression of the diffuse TBI [83]. Glutamate can synthesize the gamma-aminobutyric acid (GABA), which is a common linkage between the pathophysiology associated with secondary TBI and seizure susceptibility [84]. Metabolic conversion of glutamine can increase plasma glutamate levels [85], which can induce addition brain damage due to its excitoxic and edema-aggravating potential following severe TBI [84]. Ischemia results in BBB impairment, leading to an increased expression of glucose transportation to the brain [86]. The changes of plasma phenylalanine and branched chain amino acids (isoleucine, leucine and valine) influence intracranial pressure and jugular venous oxygen saturation in patient with severe TBI [87]. Despite these major metabolites altered in TBI, they failed to be the sensitive biomarkers of acute TBI. These results show that metabolic switch-like transitions occur in TBI between and after the period post 24 hours. Therefore, our observations may deduce a conclusion that the process of TBI are likely related to the disturbance of biological modules, which requires clusters of functionally related biomarkers to reflect the full spectrum of the response of brain tissue to TBI.

Overall, this metabolomics platform expands the potential range of research for acute TBI compared to single biomarker-based methods. There are several limitations of this study: metabolomics technologies including high-performance liquid chromatography/mass spectrometer (HPLC/MS) and nuclear magnetic resonance (NMR) methods should be applied in the future studies to further confirm the potential biomarkers of the pathophysiology of acute TBI. In addition, clinical samples should be collected from acute TBI patients to validate our findings.

## Conclusion

In summary, here we report comprehensive metabolomics profiles for identifying relevant perturbations in acute CCI rats by GC/MS. CCI induced distinctive changes in metabolites including linoleic acid metabolism, amino acid metabolism, galactose metabolism, and arachidonic acid metabolism. Specifically, the acute CCI group exhibited significant alterations in proline, phosphoric acid, β-hydroxybutyric acid, galactose, creatinine, L-valine, linoleic acid and arachidonic acid. A receiver operating characteristic curve analysis showed that the above 8 metabolites in plasma could be used as the potential biomarkers for the diagnosis of acute TBI. Furthermore, this study is the first time to identify the galactose as a biomarker candidate for acute TBI. This comprehensive metabolic analysis complements target screening for potential diagnostic biomarkers of acute TBI and enhances predictive value for the therapeutic intervention of acute TBI.

## Supporting information

S1 Table Detailed information on the raw GC/MS data. (XLSX)
